# Discordance Between Inflammatory Bowel Disease Specialists and Insurance Authorization Denials—A Survey of Specific Inflammatory Bowel Disease Treatment Scenarios

**DOI:** 10.1093/crocol/otad082

**Published:** 2023-12-30

**Authors:** Anastasia Naritsin, Neev Mehta, Randall Pellish

**Affiliations:** Department of Internal Medicine, Lahey Hospital and Medical Center, 41 Mall Rd, Burlington, MA 01805, USA; Department of Internal Medicine, Lahey Hospital and Medical Center, 41 Mall Rd, Burlington, MA 01805, USA; Department of Gastroenterology, Lahey Hospital and Medical Center, 41 Mall Rd, Burlington, MA 01805, USA

**Keywords:** inflammatory bowel disease, prior authorization denials, biologics, small-molecule drugs

## Abstract

**Background:**

Prior authorizations are generally required by insurers for gastroenterologists to prescribe biologics and small-molecule drugs to treat inflammatory bowel disease (IBD). Authorization denials occur in a wide variety of clinical scenarios, including denials of standard and nonstandard medication dosing.

**Methods:**

We performed a national cross-sectional survey on a broad variety of specific clinical scenarios to assess experience and opinions on whether or not insurance authorization denials are in accordance with clinical expertise.

**Results:**

Eighty-four gastroenterologists completed the survey. Denial experience was common for infliximab dose modifications, vedolizumab dose modifications, ustekinumab first-time therapy, and maintenance dosing. The bulk of disagreement with authorization denials involved scenarios of dose escalation and re-induction guided by both loss of clinical response and/or therapeutic drug monitoring, denial of re-authorizations of stable dosing, and use of non-anti-TNFs in specific patient populations including the elderly and patients with multiple comorbidities. Respondents unanimously agreed that insurance companies do not play an adequate role in helping patients obtain PA. Furthermore, most of the respondents agree that to decrease the burden of the PA process, peer–peer processes should be between other IBD-trained providers who understand these complex treatment strategies.

**Conclusions:**

Our cross-sectional survey highlights the degree of discordance in clinical decision-making between insurers and gastroenterologists. Further engagement between gastroenterologists and insurers is needed to foster common understanding on these discordant authorization denials in these real-world clinical IBD scenarios.

## Graphical Abstract

**Graphical Abstract:**
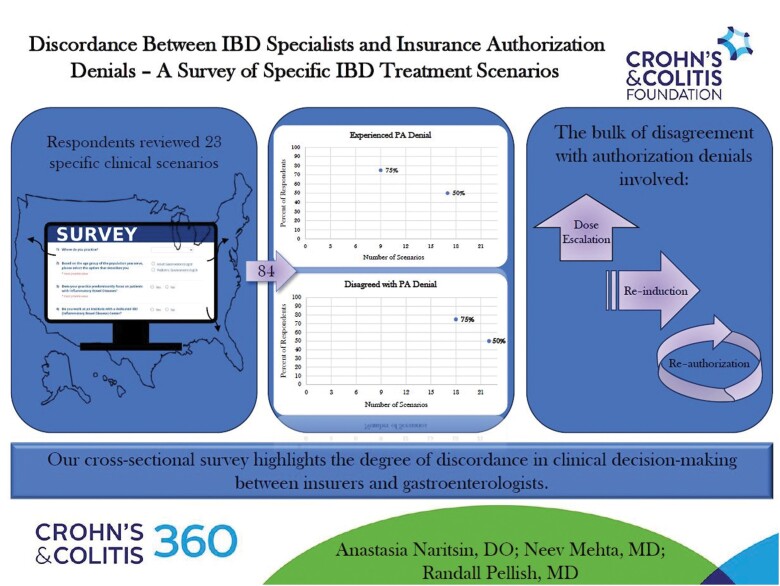

**Key Messages**

- Prior authorizations are generally required by insurers for gastroenterologists to prescribe biologics and small-molecule drugs to treat inflammatory bowel disease (IBD). Authorization denials occur in a wide variety of clinical scenarios, including denials of standard and nonstandard medication dosing.
- Our cross-sectional survey highlights the degree of discordance in clinical decision-making between insurers and gastroenterologists.
- Further engagement between gastroenterologists and insurers is needed to foster common understanding on these discordant authorization denials in these real-world clinical scenarios to prevent therapy delay and adverse events.

## Introduction

Inflammatory bowel disease (IBD), which encompasses Crohn’s disease and ulcerative colitis, affects approximately 3 million Americans and is characterized by periods of remission and relapse which can be associated with high healthcare costs from medications, surgeries, and disease complications.^1,2^ Biologics and small-molecule drugs (SMD) have revolutionized the treatment of IBD by delaying disease progression, reducing hospitalizations and surgeries, and achieving higher remission rates.^1,3^ Despite their great success, insurance plans consider biologics and SMDs “specialty drugs” and require prescribing gastroenterologists to first obtain insurance prior authorizations (PAs).^1^ Prior authorizations are cost-controlling initiatives used by insurance companies to address utilization and possible inappropriate prescribing.^3–5^ Patients and gastroenterologists are experiencing increasing authorization denials in a wide variety of IBD clinical scenarios, including denials of standard and nonstandard medication dosing directed by commonly accepted clinical indications.^1,2^ Discordance between physician-directed treatment strategies and authorization denials poses a problem for patients and physicians, and questions the utility, appropriateness, and disease impact of PA policies.^3^ Prior surveys have identified that the PA process is incredibly burdensome and requires significant resources and time allotment, and has led to delays in care, sometimes resulting in unfavorable clinical outcomes.^1,3,5,6^

Limited data exist on gastroenterologist experience with specific PA denial scenarios in IBD. Our cross-sectional survey assesses gastroenterologists’ experience on a broad variety of clinical scenarios and whether or not the authorization denials are in accordance with clinical expertise. Our objective was to gather data to define the general experience of authorization denial scenarios, opinions on the clinical appropriateness of these denials, and broad opinions on the PA process.

## Materials and Methods

A national cross-sectional survey was distributed via email and social media to over 600 practicing gastroenterologists between May and September 2022. Practicing gastroenterologists were primarily identified by searching for IBD specialists in academic medical centers from each state. The survey consisted of 23 scenarios where authorization denials are occurring, with 3 thematic domains including general experiences with the authorization denial scenarios, opinions on the clinical appropriateness of these denials, and broad trends in denial process. The survey was designed by IBD specialists who were members of the Chapter Medical Advisory Committee of the New England Chapter of the Crohn’s and Colitis Foundation based on prior personal clinical experiences with authorization denials in specific clinical scenarios. The survey utilized the Redcap data management system for data collection and analysis. The percentages for PA denial experience and disagreement were calculated solely based on the number of respondents who answered each individual scenario. Test–retest reliability was established by having a trap question that is expected to have a unanimous “no” when asked if providers have experienced this denial scenario. The study was approved by the Lahey Hospital and Medical Center Institutional Review Board.

## Results

### Demographics

Eighty-four gastroenterologists (13% of ~630 providers who received the survey) completed the survey, including 72 (85.7%) adult gastroenterologists and 12 (14.3%) pediatric gastroenterologists. Sixty-four (76.2%) respondents work in a practice that predominantly focuses on patients with IBD, and 62 (73.8%) work at an institute with a dedicated IBD Center. Respondents represented 22 states plus the District of Columbia and a variety of practice types (Table 1).

**Table 1.** Demographics of survey respondents

Measure	Item	Count	Percentage (%)
Geographic region	Northeast	43	51
	Midwest	11	13
	West	17	20
	Southeast	13	16
Population type	Adult GI	72	86
	Pediatric GI	12	14
Predominant focus on IBD	Yes	64	76
	No	20	24
Dedicated IBD center at workplace	Yes	62	74
	No	22	26

GI, gastrointestinal; IBD, inflammatory bowel disease.

### Prior Authorization Denial Scenarios

**Figure 1.**
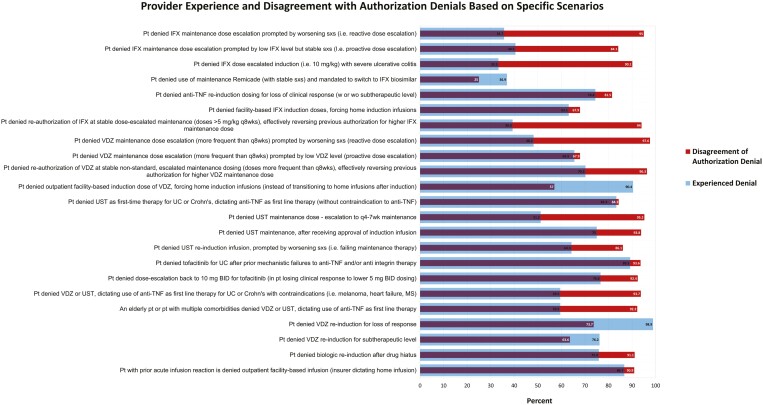
Denial experience based on scenario and provider agreement in regard to each denial.

Of the 84 respondents, >75% experienced PA denials for 9 of the scenarios, and >50% experienced PA denials for 17 of the scenarios. Furthermore, >75% disagreed with the PA denials for 18 of the scenarios, and >50% disagreed with the PA denials for 22 of the scenarios (Supplement A). Denial experience was common for infliximab dose modifications, vedolizumab dose modifications, ustekinumab first-line therapy, and ustekinumab dose modification. Most of the disagreement with authorization denials included scenarios of dose escalation and re-induction guided by both loss of clinical response and/or therapeutic drug monitoring, denial of re-authorizations of stable dosing, and use of non-anti-tumor necrosis factors as first-line therapy in specific patient populations including the elderly and patients with multiple comorbidities. In addition, 60 (71.4%) of the respondents experienced requests for escalated doses being approved at a standard dose instead, without outright denial of the medication. The specific scenarios and distribution of responses are represented in Figure 1.

### The Role of the Insurer

Eighty-three (100%) respondents reported that the insurance company does not play an adequate role in helping patients navigate the complex PA process. Fifty-five (65.5%) respondents reported that the insurance company did not provide a recommendation for an alternative evidence-based medication strategy when the requested medical treatment choice was denied by the insurer/payer. Sixty-two (73.8%) respondents believe the insurance companies/payors should play a role in organizing logistics for insurance-dictated transitions from facility-based infusions to home infusions.

### Impact of Peer–Peer Processes and Third-Party Companies

With attempts to appeal PA denials, 79 (94%) responders face multiday delays in completing a peer-to-peer process. Given the complexities of decision-making on biologics medications, 80 (95.2%) of the respondents feel it is appropriate to have a peer-to-peer review with a gastroenterologist, as opposed to a physician of a different specialty or discipline. Of the 83 respondents, 64 (77.1%) have also encountered third-party companies acting on behalf of the insurance company when dealing with biologic authorization issues, thus complicating the appeals process.

### Practice Strategies and Impact of PA Denials on Patient Care

**Figure 2.**
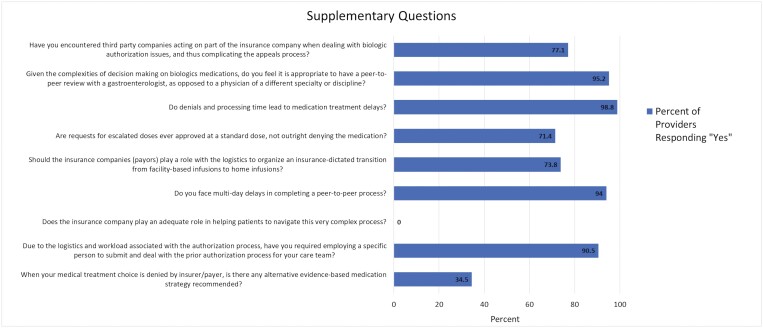
Broad trends in gastroenterologists’ experience with prior authorization denials.

Due to the logistics and workload associated with the authorization process, 76 (90.5%) of respondents have required employing a specific person to submit and deal with the prior authorization process for their care team. Furthermore, 83 (98.8%) respondents reported that PA denials and processing time lead to medication treatment delays affecting patient care (Figure 2).

## Discussion

The advent of biologics and SMDs to treat IBD has revolutionized the care of IBD patients. With such a complex disease, gastroenterologists face multiple clinical scenarios for which complex medication strategies and decisions exist. Insurer authorization denials create obstacles to how physicians treat patients and are inherently conflictual to the physicians’ clinical experience and expertise. Our survey highlights the common experience amongst gastroenterologists with PA denials and the degree of discordance in clinical decision-making between insurers and gastroenterologists.

Respondents have unanimously agreed that insurance companies do not play an adequate role in helping patients obtain PAs. There was also significant evidence of a lack of partnership between physicians and insurers to provide patients with options and navigate PA denials. Furthermore, most of the respondents agree that to decrease the burden of the PA process, peer–peer processes should be between other IBD-trained providers who understand these complex treatment strategies.

Prior surveys regarding PAs, including a 2021 survey by the American Medical Association, noted that 93% of physicians reported PAs led to delays in care, 90% saw a negative impact on clinical outcomes, and 34% experienced PA denials resulting in a serious adverse event.^3,7^ Furthermore, in cases of therapy abandonment, 87% were due to PAs, and 30% of PA requisites were rarely or never evidence-based.^7^ Studies have also shown that having an FDA-labeled indication increases the likelihood of PA approval and dose escalation requests had the lowest rates of approval, which complicates the requests for nonstandard dosing for patients who experience partial or loss of response to therapy.^7^

Navigating the PA process is burdensome. Bhat et al. reported on resources that can improve the PA process including hiring a clinical pharmacist, identifying rules of common payers, utilizing electronic PAs, obtaining copies of payer formularies, obtaining reliable points of contact, and improving patient documentation. Furthermore, if a PA is denied, Bhat et al. suggested emphasizing the aggressiveness of the patient’s disease and consequences of ineffective therapy, stressing the high cost of adverse events, emphasizing patient-centered therapy selection, providing notes and clinical data, and referring to society guidelines.^8^ To further advocate for patients, multiple bills have been introduced to protect patients in step therapy protocols, including requiring payers to respond within a certain timeline.^8^

A strength of this study is the involvement of gastroenterologists from practices across the United States. Our limitations include a low overall response rate limiting generalizability, recall bias that might impact a respondents report on their personal experience with each scenario, and the potential for selection bias as providers who experience high PA burden may be more inclined to respond. The survey did not include clinical scenarios with newer medications such as upadacitinib and risankizumab as the survey was created prior to FDA approval of these medications. Prior surveys on PA process have raised similar bias concerns.^9^

Multiple specific clinical scenarios exist where authorization denials create obstacles in IBD treatment. Our survey highlights the common experience amongst gastroenterologists with authorization denials and the degree of discordance in clinical decision-making between insurers and gastroenterologists. Further engagement between gastroenterologists and insurers is needed to foster common understanding on these discordant authorization denials in these real-world clinical scenarios.

## Supplementary Material

otad082_suppl_Supplementary_Figure

otad082_suppl_Supplementary_Appendix

## Data Availability

Data available in Supplementary Material. Some data not publicly available.
