# Supplementary figures and images for: Discordance Between Inflammatory Bowel Disease Specialists and Insurance Authorization Denials—A Survey of Specific Inflammatory Bowel Disease Treatment Scenarios
Source: Crohns Colitis 360. 2023 Dec 30;6(1):otad082. doi: 10.1093/crocol/otad082 (PMC10805526; doi:10.1093/crocol/otad082)

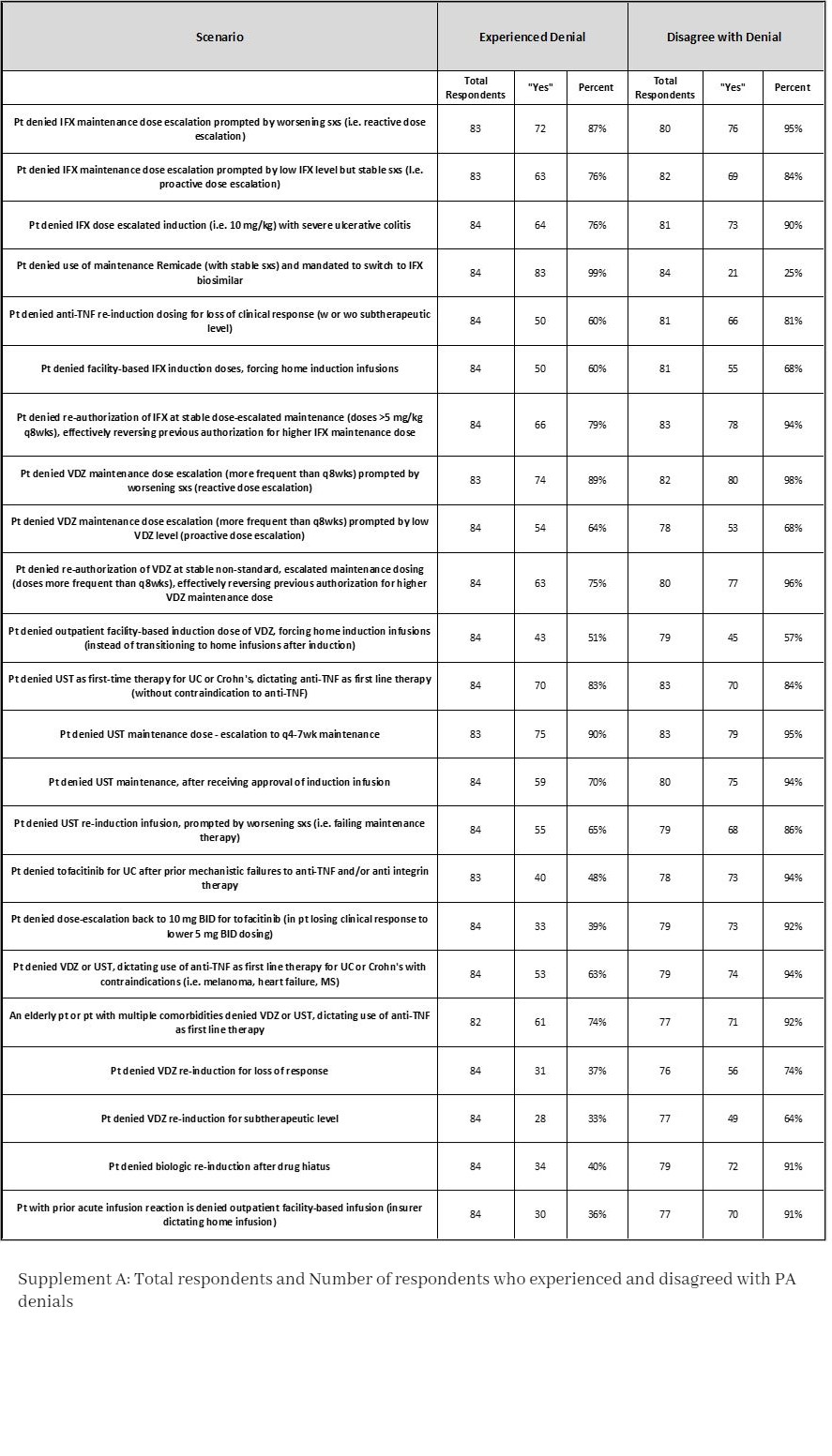

Supplement: otad082_suppl_Supplementary_Figure [file otad082_suppl_supplementary_figure.jpeg]
