# Supplementary material for: Discordance Between Inflammatory Bowel Disease Specialists and Insurance Authorization Denials—A Survey of Specific Inflammatory Bowel Disease Treatment Scenarios
Source: Crohns Colitis 360. 2023 Dec 30;6(1):otad082. doi: 10.1093/crocol/otad082 (PMC10805526; doi:10.1093/crocol/otad082)
Supplement: otad082_suppl_Supplementary_Appendix [file otad082_suppl_supplementary_appendix.docx]

**Appendix:**

Respondents expressed frustrations with the PA process, including statements of “I am tempted to shy away from IBD patients because they require much more work than other patients with regards to any changes made to treatment” and that “clinic staff spend so much time on this that it affects the ability to do their regular nursing and admin work … with staff contemplating leaving over the dissatisfaction of the PA process”. One respondent indicated that the PA process is “the biggest pain point for caring for IBD patients ... [being] incredibly frustrating and time-consuming” and is “the single biggest source of stress and burnout in [their] practice … [keeping them] from being the physician [they] want to be, and [their] patients need [them] to be”. A respondent indicated they “would like an option to have a peer-to-peer review with a gastroenterologist with advanced training in IBD" given that “peer-to-peer review with a gastroenterologist is not sufficient, as most gastroenterologists who do this kind of insurance work do not understand management of complex IBD”. Another respondent noted that “a significant amount of harm has occurred to numerous patients at [their] facility due to insurance denials and delays of appropriate therapies because of these denials”, leading to “dozens of flares and multiple disability claims in [their] patients over the past 18 months”.
